# The Role of Illness Perception and Its Association With Posttraumatic Stress at 3 Months Following Acute Myocardial Infarction

**DOI:** 10.3389/fpsyg.2018.00941

**Published:** 2018-06-07

**Authors:** Mary Princip, Christina Gattlen, Rebecca E. Meister-Langraf, Ulrich Schnyder, Hansjörg Znoj, Jürgen Barth, Jean-Paul Schmid, Roland von Känel

**Affiliations:** ^1^Division of Cardiovascular Prevention, Rehabilitation and Sports Medicine, Department of Cardiology, Bern University Hospital, Inselspital and University of Bern, Bern, Switzerland; ^2^Clienia Schlössli AG, Oetwil am See, Zurich, Switzerland; ^3^University of Zurich, Zurich, Switzerland; ^4^Department of Clinical Psychology and Psychotherapy, University of Bern, Bern, Switzerland; ^5^Complementary and Integrative Medicine, University of Zurich, Zurich, Switzerland; ^6^Department of Cardiology, Clinic Barmelweid, Barmelweid, Switzerland; ^7^Department of Neurology, Bern University Hospital, Inselspital and University of Bern, Bern, Switzerland; ^8^Department of Consultation-Liaison Psychiatry and Psychosomatic Medicine, University Hospital Zurich, Zurich, Switzerland

**Keywords:** llness perception, posttraumatic stress disorder (PTSD), myocardial infarction patients, brief illness perception questionnaire, heart attack

## Abstract

**Background:** The aim of this study was to investigate the relationship between illness perception and posttraumatic stress disorder (PTSD) symptoms at three months following acute myocardial infarction (MI).

**Methods:** Patients (*n* = 96) were examined within 48 h and 3 months after the illness episode. The brief revised illness perception questionnaire (Brief-IPQ) was used to assess patients' cognitive representation of their MI. At 3-month follow-up, the Posttraumatic Diagnostic Scale (PDS) and the Clinician-Administered PTSD Scale (CAPS) were used to assess the level of PTSD symptoms.

**Results:** The subjective perception of the illness, including higher harmful consequences (*r* > 0.35, *p* < 0.01), higher illness concerns (*r* > 0.24, *p* < 0.05) and more emotional impairment (*r* > 0.23, *p* < 0.05), was associated with both self-rated and clinician-rated PTSD symptoms. Beliefs regarding harmful consequences after acute MI were independently associated with levels of PTSD symptoms assessed with both the self-rated PDS and CAPS interview (standardized β coefficient = 0.24; *P* < 0.05) adjusted for demographic factors, cognitive depressive symptoms, fear of dying during MI, factors related to study design, and illness severity.

**Conclusions:** The findings suggest that initial perception of acute MI is significantly associated with PTSD symptoms attributable to MI at 3 months follow-up.

## Introduction

Myocardial infarction (MI) is one of the leading causes of death in the developed world (White and Chew, 2008). According to recent estimates, ~6 million people die annually due to cardiovascular diseases, including coronary heart disease (Leal et al., 2006). Patients usually perceive an acute MI as a sudden and life-threatening event, involving high intensity of fear of dying, helplessness and loss of control. Two reviews showed that ~15% of patients develop Posttraumatic Stress Disorder (PTSD) after an acute MI (Gander and von Känel, 2006; Edmondson et al., 2012). It should be noted that this previous research applied Diagnostic and Statistical Manual of Mental Disorders (DSM)-IV criteria for the assessment of PTSD (American Psychiatric Association, 2000), whereas PTSD diagnostic criteria were recently revised in the DSM-5 (American Psychiatric Association, 2013). According to the DSM-5 the diagnosis of PTSD requires several criteria: Criterion A stipulates exposure to a traumatic or stressful event, leading to Criterion B, re-experience of the event in form of e.g., nightmares and flashbacks; Criterion C, avoidance of event-related stimuli; Criterion D, persistence of negative feelings and thoughts, e.g., about oneself or the world that began or worsened after the event; and Criterion E, trauma-related arousal and reactivity that began or worsened after the trauma, such as hyperarousal, risky behavior, or aggression. Symptom criteria have to be present for at least 1 month (Criterion F) and must significantly interfere with the patients' daily functioning (Criterion G). Symptoms of PTSD after MI increase the risk of hospital readmission, recurrent MI and all-cause mortality (Edmondson et al., 2012) and are also associated with poor quality of life and general health, adverse health behaviors and medical comorbidities (Edmondson and von Kanel, 2017). In addition, PTSD is associated with poor adherence with cardiac rehabilitation cardiac medication (Kubzansky and Koenen, 2007; Kubzansky et al., 2007). Younger age, as well as helplessness, pain and fear of dying during MI (Hari et al., 2010), lower educational level (Ginzburg et al., 2002) and depressive symptoms (Whitehead et al., 2005) were shown to be predictive for PTSD symptoms in the first year after acute MI.

Following the self-regulatory model (Leventhal, 1984), illness recovery also depends on how patients view their illness and on their ability to cope with the new situation. Individuals are considered to master a threat to their health, such as acute MI, by developing their own appraisal, and determining subsequent coping procedures, which then modify illness outcome. The self-regulatory model integrates a constant feedback loop, where consequences of appraisal processes are fed back into the structure of illness perception and coping reactions. Illness perception has been described to consist of five cognitive components (causes of the illness, identity, consequences of the illness, time line and ways to control or cure the illness). Taken all five beliefs together, an illness schema is formed which determines how patients respond to their illness (Leventhal, 1984; Broadbent et al., 2006b; O'Connor et al., 2008). A number of studies have shown that illness perception predicts health behaviors (e.g., treatment adherence, functional outcomes) (Broadbent et al., 2004, 2009b; Reynolds et al., 2007), may increase the risk of PTSD symptoms (Oflaz, 2014) and affect recovery from MI (Petrie et al., 1996; Broadbent et al., 2006a; French et al., 2006). Petrie et al. showed that patients' perceptions of their MI had important effects on different aspects of recovery such as social interaction, recreational and sexual activity, resumption of work, and general domestic tasks (Petrie et al., 1996, 2002; Juergens et al., 2010). Furthermore, maladaptive negative cognitions about the consequences of acute MI, belief in a longer timeline of their condition, and lower cure and control beliefs, predicted depression (Dickens et al., 2008a,b). Recent research showed that MI patients' negative illness beliefs as well as anxiety in their spouses can be reduced by a brief illness perception intervention (Broadbent et al., 2009a). The time after an acute MI was shown to be a period of vulnerability after which patients may not be able to return to normal functioning if they fail to successfully cope with the traumatic experience (Daly et al., 2000; Chung et al., 2008; Lowe et al., 2010). There are two studies investigating the association between illness perception after an acute MI and PTSD symptoms (Sheldrick et al., 2006; Oflaz, 2014). In the present study we addressed this topic whereby focusing on patients with a high level of distress during MI, for whom adaptation to MI-related experiences may be particularly challenging. Furthermore, we measured illness perception shortly, i.e., within 48 h, after MI.

Our primary aim was to examine the predictive value of illness perception for the development of MI-induced PTSD symptoms at 3 months follow-up. We hypothesized that patients who perceive their MI with a high level of distress would show more self-rated and clinician-rated PTSD symptoms. The second aim was to identify the strongest associations of PTSD symptomatology with the different dimensions of illness perception. Finally, we aimed to examine if the association between illness perception and PTSD symptoms would be independent of demographic, psychological and medical variables as potential covariates.

## Methods

### Study participants and design

The data for the present analysis was collected between January 2013 and March 2015. A total of 130 patients with acute MI, admitted to the coronary Care unit (CCU) of a tertiary university center (Bern University Hospital, Inselspital), were included in the Myocardial Infarction Stress Prevention Intervention (MI-SPRINT) randomized controlled trial (Meister et al., 2013). The aim of MI-SPRINT was to reduce the incidence of PTSD symptoms by using an early behavioral intervention. Eligibility criteria for the trial were age 18 years or older, confirmed acute ST-segment elevation MI (STEMI) or non-ST-segment elevation MI (NSTEMI), stable hemodynamic conditions, sufficient knowledge of German, positive distress screening at admission, i.e., scores of at least 5 points on a numeric rating scale, ranging from 0 to 10, for chest pain and for fear of dying and/or helplessness, no serious comorbid diseases causing death within 1 year, no cognitive impairment, no current severe depression, no suicidal ideations. Patients participating in another trial at the department of cardiology, patients with suicidal intentions in the last 2 weeks and patients who underwent emergency coronary artery bypass grafting were excluded. After written informed consent had been obtained, patients' illness perception was assessed within 48 hours after the acute coronary event. Patients were then randomized to a 45-minute single-session intervention of either trauma-focused counseling (*verum* group), targeting potential MI-induced posttraumatic reactions, or an active control intervention (control group), targeting the general role of psychosocial stress in patients with heart disease. Three months after the counseling session, patients' PTSD symptoms were assessed. Of the 130 patients, 70 were in the *verum* group and 60 were in the control group. Patients' educational level was categorized across four groups: “Lower than apprenticeship or vocational school,” “Apprenticeship or vocational school,” “High school graduation,” and “University graduation (including applied sciences).”

### Illness severity

We obtained all the medical variables necessary to compute the prognostic Global Registry of Acute Coronary Events (GRACE) score (Elbarouni et al., 2009; Bhattacharyya et al., 2010). The score provides an objective prognostic marker of illness severity and was calculated with the GRACE ACS (Global Registry of Acute Coronary Events; Acute Coronary Syndrome) Risk and Mortality Calculator (http://www.mdcalc.com/grace-acs-risk-and-mortality-calculator). It comprises 8 variables (i.e., age, heart rate, systolic blood pressure, plasma creatinine concentration, Killip classification, cardiac arrest, and STEMI or NSTEMI) and is a scoring system to prospectively estimate the probability of death within 6 months after admission for an acute coronary syndrome. Although elevated creatinine kinase isoenzyme MB (CK-MB) levels are part of the GRACE score, we separately considered the absolute CK-MB peak value as a measure of cardiac injury.

### Psychometric assessment

#### Illness perception

The brief German version of the self-rated Illness Perception Questionnaire (Brief IPQ) was used to assess patients' cognitive representations of their MI (Broadbent et al., 2006b). The Brief IPQ consists of 9 items. These dimensions are “consequences,” “time-line,” “personal control,” “treatment control,” “identity,” “illness concern,” “coherence,” and “emotional representation.” We applied the brief version of the IPQ to limit questionnaire burden for patients in the acute medical setting.

The ninth item is a causal item, where patients are asked to write down three self-assumed causes for their MI. This item was not used for the current analysis. Each item is scored between 0 and 10. Item 1 for example asks: “*How much does your illness affect your life?*” 0 means *not at all*, 10 means *severely affects my life*. The reliability was acceptable in this sample (Cronbach's α = 0.74).

#### Depressive symptoms

The Beck Depression Inventory second edition (BDI-II) (Ahrari et al., 2013) is an instrument with two subscales, cognitive/affective and somatic/affective symptoms of depression as potential predictors of PTSD symptoms. We used the validated German version of the BDI-II (Kühner et al., 2007). Again, in order to limit questionnaire burden for patients, we only used the cognitive symptom subscale. The validated German version of the BDI-II (Kühner et al., 2007) cognitive subscale consists of 13 items. Each item is rated on a Likert scale ranging from 0 to 3 (total score 0–39). The BDI-II cognitive subscale has been applied to patients with medical conditions including MI (Poole et al., 2009). Ratings for symptom severity are 0–3 (no depressive symptoms), 4–6 (mild symptoms), 7–9 (moderate symptoms), > 10 (clinically relevant symptoms). The reliability of the cognitive symptom subscale was acceptable in this sample (Cronbach's α = 0.71).

#### Posttraumatic stress

To assess PTSD symptoms at 3 months follow-up, we used the validated German version of the Clinician-Administered PTSD Scale (CAPS) (Blake et al., 1995) and the self-rated Posttraumatic Diagnostic Scale (PDS) (Ehlers et al., 1996; Foa et al., 1997). The study was planned before the introduction of the DSM-5, so the CAPS and PDS both refer to DSM-IV diagnostic criteria for PTSD. The CAPS interview assesses six criteria of PTSD (A-F). The A Criterion designates exposure to a traumatic event, which involves risk or actual physical injury and emotional response to the event (intense fear, horror or helplessness). Re-experiencing symptoms (Criterion B), avoidance symptoms (Criterion C), and hyperarousal symptoms (Criterion D) together cover the 17 DSM-IV PTSD symptoms that are quantified on a 5-point Likert-type scale for frequency and intensity. The scale ranges from “never” (0) to “daily or almost daily” (4) and from “none” (0) to “extreme” (4). A particular symptom is present if frequency is scored with at least 1 point and intensity with at least 2 points. In total, a sum score of 136 can be reached. To diagnose PTSD, Criterion B requires 1 out of 5 symptoms, Criterion C 3 out of 7 symptoms and Criterion D 2 out of 5 symptoms all of which must have been lasted for at least 1 month (Criterion E). Impairment in daily functioning after the traumatic event is also required (Criterion F). Like Blanchard et al. (1995) we diagnosed sub-threshold PTSD when criteria B, E, and F plus either Criterion C or Criterion D were fulfilled. In the present sample, the CAPS sum score showed good internal consistency (Cronbach's α = 0.80), whereas consistency for the three subscales was poorer (re-experience, Cronbach's α = 0.70; avoidance, Cronbach's α = 0.68; arousal, Cronbach's α = 0.49).

The Posttraumatic Stress Diagnostic Scale (PDS) is a 17-item self-report questionnaire that is also based on DSM-IV criteria for PTSD (Foa et al., 1997). On a 4-point scale, participants rate the presence of symptoms in the previous month from “not at all” (0) to “very much” (3). We categorized symptom severity per the following cut-offs: 0 (no symptoms), 1–10 (mild symptoms), 11–20 (moderate symptoms), 21–35 (moderate-to-severe symptoms) and >36 (severe symptoms). To fulfill PTSD caseness criteria, 1 item of re-experiencing, 3 items of avoidance and 2 items of arousal must be present for at least 1 month. We used the validated German version of the PDS (Ehlers et al., 1996). Internal consistency of the scale was good in our sample (re-experiencing, Cronbach's α = 0.81; avoidance, α = 0.80; arousal, α = 0.82; sum score: α = 0.90).

#### Data analysis

Statistical analysis was carried out with SPSS Version 22.0 statistical software package (SPSS, Inc., Chicago, USA). All tests were two-tailed with significance level at *P* < 0.05. Data were verified for normal distribution using the Kolmogorov-Smirnov Test. To approximate a normal distribution, values for the CAPS sum score were subject to square root transformation before analysis.

Group differences were calculated using Student's *t-*test for normally distributed variables and Mann-Whitney *U* test for non-parametric variables. Bivariate correlations between two variables were estimated with Pearson coefficients. We then applied hierarchical linear regression analysis with forced entry of covariates, added in five blocks, to compute the independent association of illness perception dimensions with sum scores of both clinician- and self-rated PTSD symptoms. Covariates were selected a priori based on previous literature on a potential association with PTSD symptoms (Edmondson et al., 2012); covariates included demographic factors (gender, age, educational level), peritraumatic stress (fear of dying during MI), depressive symptoms and illness severity (GRACE risk score, peak CK-MB level). In addition, as patients received a single psychological counseling intervention session (*verum* vs. control group), we firstly adjusted for the variable “intervention” (*verum* vs. control group) to investigate the association between illness perception and PTSD symptoms, independent of the type of intervention. Secondly, an interaction effect between “intervention” (*verum* vs. control group) and illness perceptions (“consequences”) was examined. The first block consisted of the demographic variables. The second block consisted of psychological variables (fear of dying during MI, depressive symptoms, intervention) and the third block of objective measures of MI severity. The fourth block consisted of the illness perception dimension “consequences” and the fifth and last block of the interaction effect between “intervention” and “consequences.” Effect sizes are reported as standardized *B* coefficients. Data output was verified for absence of multicollinearity by Durban Watson statistics and linearity of residuals was verified by means of scatter plots.

## Results

### Patient characteristics

The mean age ± SD of participants was 60.3±10.4 years. All participants were Caucasian (*n* = 130) and the majority were men (81.5%). At the time of their MI, 43.8% worked full time, 12.3% worked part time and 43.8% were retired or unemployed; 11.5% of participants had been diagnosed with a previous MI. 63.8% were married, 16.9% were divorced, 5.4% were widowed and 13.8% were single. 28.5% of the participants lived alone and 71.5% were cohabiting. The majority of the patients had completed “vocational school” (*n* = 89) or an “apprenticeship.” The average hospital stay was 3.7 ± 2.1 days. Of the 130 patients, 47.9% (*n* = 92) had STEMI and 29.2% (*n* = 38) had NSTEMI. All of the patients had undergone coronary angioplasty; 92.3% (*n* = 120) were treated with at least one drug-coated stent. Thirty four patients did not attend the 3-month follow-up appointment because of lack of interest, death or change of contact information such that from the originally enrolled 130 patients, 96 could be assessed at follow-up (attrition rate 26.2%).

### Prevalence of clinically relevant PTSD symptomatology

Clinician ratings yielded a prevalence of 1% for full PTSD and of 14.4% for clinically relevant PTSD when also including subthreshold PTSD. The prevalence of clinically relevant PTSD symptom levels was18.8% based on self-ratings. Table 1 summarizes the scores of the psychological and physiological assessments. PTSD symptom levels did not significantly differ by gender and age regarding sum scores and scores for subscales for either the CAPS interview or PDS. Older patients reported fewer re-experiencing (*r* = −0.21; *P* < 0.05) and hyperarousal (*r* = −0.20; *P* < 0.05) symptoms in the CAPS interview, whereas there was no significant relation between age and the three PTSD symptom clusters of the PDS. No significant relationship between educational level and PTSD symptomatology was found. Patients who dropped out did not significantly differ from the final sample in terms of sex, education, previous MI, previous PTSD and illness perception dimensions. Also, there was no significant difference in PTSD symptoms between patients with and those without a history of previous MI.

**Table 1:** Mean scores for psychological and physiological scores.

	**Patients**
Fear of dying	5.51 (2.60)
Helplessness	5.63 (2.41)
Pain	8.11 (1.56)
Beck Depression Inventory-II	2.88 (2.90)
**POSTTRAUMATIC DIAGNOSTIC SCALE**	
Re-experiencing	1.45 (2.13)
Avoidance	2.08 (2.99)
Arousal	2.40 (2.80)
Sum score	5.78 (6.64)
**CLINICIAN-ADMINISTERED PTSD SCALE**	
Re-experiencing	3.36 (4.58)
Avoidance	3.57 (4.86)
Hyperarousal	5.30 (4.80)
Sum score	12.28 (11.63)
**ILLNESS PERCEPTIONS**	
Consequences	4.75 (2.87)
Timeline	6.42 (3.34)
Personal control	4.28 (2.31)
Treatment control	2.14 (1.87)
Identity	3.29 (2.41)
Illness concern	4.91 (2.97)
Coherence	3.74 (2.40)
Emotional representation	3.89 (2.83)
Creatine kinase isoenzyme MB	151.73 (162.99)
Grace risk score	106.27 (26.29)

*Data are presented as mean (SD); PTSD, Posttraumatic Stress Disorder*.

### Brief IPQ characteristics

There were gender differences in Brief IPQ items for the subscale “control over the illness,” with men reporting higher perceptions of control than women [*t*_(97)_ = 2.75; *P* < 0.007]. Older patients had less illness concerns (*r* = 0.26; *P* < 0.01) and assumed that the illness would last for a shorter period of time (*r* = −0.25; *P* < 0.01) than younger ones. Patients with higher educational level reported lower personal control (*r* = −0.20; *P* < 0.05) and longer illness duration (*r* = 0.20; *P* < 0.05).

Significant correlations emerged amongst Brief IPQ subscales “consequences,” “illness concern” and “emotional representation.” Patients with a greater belief of serious consequences after MI also had more illness concerns (*r* = 0.56; *P* < 0.001) and felt more emotionally affected (*r* = 0.39; *P* < 0.001). The stronger the patients' beliefs of having personal control over their illness, the lower was the perceived severity of consequences (*r* = −0.28; *P* = 0.001).

### Depressive characteristics

Depressive symptom levels did not significantly differ depending on age, gender, educational level and illness severity measured by the GRACE score and peak levels of CK-MB. Of the 130 participants, 97 patients (74.7%) reported no depressive symptoms, 22 patients (16.8%) had mild depressive symptoms, 8 patients (6.1%) reported moderate symptoms, and 3 patients (2.4%) had severe depressive symptoms. Thus, 11 (8.5%) patients reported clinically relevant depressive symptoms at admission.

### Bivariate associations of brief IPQ factors with PTSD symptoms

Table 2 shows that illness perception correlated with several clinician- and self-rated PTSD symptom dimensions at 3 months post-MI. Item 1 “consequences” showed significant associations with all PTSD scales. Illness concerns and emotional representation were significantly related to sum scores of both clinician- and self-rated PTSD symptoms. The CAPS sum score showed significant correlations with item 1 “consequences,” item 2 “timeline,” item 6 “illness concern” and item 8 “emotional representation,” whereas the PDS sum score significantly related to item 1 “consequences,” item 5 “identity,” item 6 “illness concern” and item 8 “emotional representation.” Personal control and treatment control as well as the coherence of the illness showed almost no significant relationship with PTSD symptoms. Table 3 shows the associations between illness perception dimensions and PTSD symptom sum scores for the *verum* and active control intervention groups separately. Similar results were found for the two groups as in the total sample, with, however, item 2 “timeline” no longer showing a significant correlation in either group.

**Table 2:** Associations between illness perception dimensions at admission and PTSD symptoms (subscales and sum scores) at 3 months follow-up after acute myocardial infarction.

	**1**	**2**	**3**	**4**	**5**	**6**	**7**	**8**
**CLINICIAN-RATED PTSD SYMPTOMS**								
CAPS re-experiencing	0.33^**^	0.16	−0.19	−0.07	0.17	0.20^*^	0.14	0.16
CAPS avoidance	0.27^**^	0.11	−0.11	0.11	0.19	0.12	0.29^**^	0.14
CAPS arousal	0.25^*^	0.25^*^	−0.13	0.08	0.16	0.21^*^	0.06	0.15
CAPS sum score	0.35^**^	0.20^*^	−0.15	0.07	0.20	0.24^**^	0.16	0.23^*^
**SELF-RATED PTSD SYMPTOMS**								
PDS re-experiencing	0.35^**^	0.13	−0.20^*^	−0.04	0.20^*^	0.26^*^	0.15	0.12
PDS avoidance	0.23^*^	0.70	−0.11	−0.18	0.31^**^	0.12	0.16	0.16
PDS arousal	0.31^**^	0.11	−0.18	0.06	0.33^**^	0.16	0.17	0.30^**^
PDS sum score	0.37^**^	0.14	−0.17	0.01	0.31^**^	0.23^*^	0.16	0.21^*^

* *P < 0.05*;

** *P < 0.01; Dimensions of the BIPQ-R (Brief Illness Perception Questionnaire-Revised) 1 = Consequences; 2 = Timeline; 3 = Personal Control; 4 = Treatment Control; 5 = Identity; 6 Illness Concern; 7 = Coherence; 8 = Emotional Representation; CAPS = Clinician-Administrated Posttraumatic Stress Disorder Scale; PDS (Posttraumatic Diagnostic Scale); PTSD (Posttraumatic Stress Disorder). Data are given as Pearson correlation coefficients for the associations of self- and interviewer-rated PTSD symptom subscales and sum scores with each dimension (1–8) of the BIPQ-R*.

**Table 3:** Associations between illness perception dimensions at admission and PTSD symptoms (sum scores) at 3 months follow-up separated for the *verum* and control intervention groups.

	***Verum*** **group (*****n*** = **50)**		**Control group (*****n*** = **46)**	
	**CAPS sum score**	**PDS sum score**	**CAPS sum score**	**PDS sum score**
Consequences	0.28^*^	0.33^*^	0.40^**^	0.39^*^
Timeline	0.12	0.12	0.28	0.18
Personal control	−0.05	−0.01	−0.23	−0.35^*^
Treatment control	−0.12	−0.04	−0.23	−0.13
Identity	0.24	0.41^**^	0.14	0.14
Illness concern	0.13	0.31^*^	0.35^*^	0.26
Coherence	0.25	0.23	0.05	0.07
Emotional representation	0.26^*^	0.39^**^	0.29^*^	0.18

* *P < 0.05*;

** *P < 0.01; CAPS sum score = Sum score of the Clinician-Administrated Posttraumatic Stress Disorder Scale; PDS sum score = sum score of the Posttraumatic Diagnostic Scale; PTSD (Posttraumatic Stress Disorder). Data are given as Pearson correlation coefficients for the associations of clinician- and self-rated PTSD symptom sum scores with each dimension (1-8) of the Brief Illness Perception Questionnaire-Revised for each group separately*.

### Associations of PTSD symptoms with other variables

Fear of dying was related to the sum score of PTSD symptoms from the CAPS interview (*r* = 0.28; *P* = 0.007) and the PDS (*r* = 0.32; *P* = 0.001), whereas helplessness and pain intensity during MI showed no such significance. Moreover, CK-MB was significantly associated with the CAPS sum score (*r* = 0.21; *P* < 0.05), whereas the prognostic GRACE Score was not significantly associated with PTSD symptomatology. In addition, Table 4 shows significant direct correlations between depressive symptoms (BDI-II) and PTSD symptoms.

**Table 4:** Associations between depressive symptoms at admission and PTSD symptoms (subscales and sum scores) at 3 months follow-up after acute myocardial infarction.

	**Depressive symptoms (BDI-II)**
**CLINICIAN-RATED PTSD SYMPTOMS**	
CAPS re-experiencing	0.16
CAPS avoidance	0.24^*^
CAPS arousal	0.22
CAPS sum score	0.28^*^
**SELF-RATED PTSD SYMPTOMS**	
PDS re-experiencing	0.23^*^
PDS avoidance	0.35^**^
PDS arousal	0.44^**^
PDS sum score	0.43^**^

* P < 0.05;

** *P < 0.01; BDI-II, Beck Depression Inventory—second edition; CAPS, Clinician-Administrated Posttraumatic Stress Disorder Scale; PDS, Posttraumatic Diagnostic Scale; PTSD, Posttraumatic Stress Disorder. Data are given as Pearson correlation coefficients for the associations of clinician- and self-rated PTSD symptom subscales and sum scores with the level of cognitive depressive symptoms*.

### Association of individual brief IPQ factors with PTSD symptoms

Item 1 “consequences” was the only independent predictor for PTSD sum scores from the CAPS interview and the PDS. The adjusted R^2^ value for the model with the CAPS sum score was 11% for item 1 “consequences” and 10% for all Brief IPQ items together (Table 5; models a and b). Similar associations emerged for the PDS sum score (not shown in detail).

**Table 5:** Association of illness perception dimensions with clinician-rated PTSD symptoms (sum score) at 3 months follow-up after myocardial infarction.

**Statistics of entire model**	**Entered variables**	***B***	**S.E**.	**β**	***P***	***r^2^***
*Model a F*_1, 94_ = 13.00, *P* < 0.001, adjusted *R*^2^ = 0.112	Consequences	0.228	0.063	0.349	0.001	0.122
*Model b F*_8, 87_ = 2.356, *P <* 0.05, adjusted *R*^2^ = 0.102	Consequences	0.165	0.080	0.252	0.044	0.046
	Timeline	0.048	0.070	0.080	0.496	0.005
	Personal control	−0.090	0.091	−0.102	0.328	0.011
	Treatment control	0.129	0.112	0.119	0.253	0.015
	Identity	−0.020	0.103	−0.024	0.849	0.000
	Concern	0.028	0.103	0.041	0.786	0.001
	Coherence	0.105	0.081	0.131	0.196	0.019
	Emotional response	0.073	0.079	0.110	0.453	0.007

*PTSD, Posttraumatic Stress Disorder; hierarchical regression model with illness perception dimensions as predicting variables of the clinician–rated PTSD symptom sum score; data from the hierarchical linear regression analysis are given as regression coefficient B with Standard Error (S.E.) and β, standardized β coefficient; predictor variables were entered in two blocks (Models a and b)*.

### Comparison of associations with posttraumatic stress at 3-month follow-up

Table 6 shows the results of the hierarchical linear regression analysis with five models (a-e), in which five blocks of covariates were linked to the CAPS sum score. There was no significant difference between the *verum* and control group regarding associations with the level of clinician-rated PTSD symptoms 3 months after MI. Sociodemographic variables and depressive symptoms showed no significant association in any model, whereas fear of dying showed significance in the second model (b), but not in the additionally adjusted models (c-e). Also, measures of illness severity per CK-MB peak values and the GRACE risk score did not emerge as significant correlates of clinician-rated PTSD symptoms.

**Table 6:** Correlates of clinician-rated PTSD symptoms (sum score) at 3 months follow-up after acute myocardial infarction.

**Statistics of entire model**	**Entered variables**	***B***	**S.E**.	**β**	***P***	***r^2^***
*Model a*	Age	−0.030	0.020	−0.170	0.137	0.028
*F*_3, 92_ = 1.641, *P =* 0.187, adjusted *R*^2^ = 0.024	Gender	−0.048	0.473	−0.012	0.920	0.001
	Educational level	−0.372	0.237	−0.177	0.120	0.031
*Model b*	Age	−0.023	0.019	−0.131	0.230	0.019
*F*_3, 89_ = 2.886, *P <* 0.05, adjusted *R*^2^ = 0.127	Gender	−0.392	0.460	−0.094	0.397	0.001
	Educational level	−0.308	0.229	−0.147	0.183	0.024
	Fear of dying	0.173	0.071	0.295	0.017	0.076
	Depression (BDI–II)	0.092	0.062	0.169	0.140	0.029
	Intervention (*verum* vs. control group)	0.085	0.386	0.025	0.826	0.001
*Model c*	Age	−0.042	0.026	−0.237	0.105	0.037
*F*_2, 87_ = 3.042, *P =* 0.055, adjusted *R*^2^ = 0.173	Gender	−0.075	0.467	−0.018	0.874	0.001
	Educational level	−0.291	0.224	−0.139	0.198	0.023
	Fear of dying	0.138	0.070	0.236	0.053	0.052
	Depression (BDI–II)	0.099	0.060	0.181	0.107	0.036
	Intervention (*verum* vs. control group)	0.051	0.385	0.015	0.894	0.001
	Peak of CK–MB	0.002	0.001	0.203	0.073	0.044
	Grace score	0.013	0.010	0.178	0.218	0.021
*Model d*	Age	−0.025	0.027	−0.140	0.350	0.013
*F*_1, 86_ = 3.312, *P* < 0.05, adjusted *R*^2^ = 0.211	Gender	−0.029	0.457	−0.007	0.949	0.001
	Educational level	−0.287	0.219	−0.136	0.195	0.024
	Fear of dying	0.128	0.069	0.218	0.067	0.047
	Depression (BDI–II)	0.076	0.060	0.139	0.212	0.023
	Intervention (verum vs. control group)	0.192	0.382	0.057	0.617	0.004
	Peak of CK–MB	0.002	0.001	0.176	0.114	0.035
	Grace score	0.006	0.011	0.086	0.562	0.005
	Consequences	0.142	0.068	0.240	0.042	0.059
*Model e*	Age	−0.025	0.027	−0.141	0.345	0.013
*F*_1, 85_ = 3.064, *P* < 0.005, adjusted *R*^2^ = 0.209	Gender	−0.057	0.458	−0.014	0.902	0.001
	Educational level	−0.316	0.221	−0.150	0.158	0.028
	Fear of dying	0.117	0.070	0.200	0.097	0.040
	Depression (BDI–II)	0.083	0.061	0.152	0.176	0.027
	Intervention (*verum* vs. control group)	0.150	0.385	0.045	0.698	0.002
	Peak of CK–MB	0.002	0.001	0.177	0.112	0.036
	Grace score	0.006	0.011	0.081	0.582	0.004
	Consequences	0.142	0.068	0.240	0.041	0.060
	Intervention x Illness perceptions	0.118	0.125	0.098	0.350	0.013

*BDI–II, Beck Depression Inventory–second edition; CK–MB, creatine kinase isoenzyme MB; PTSD, Posttraumatic Stress Disorder; data from the hierarchical linear regression analysis are given as regression coefficient B with Standard Error (S.E.) and β, standardized β coefficient; predictor variables were entered in five blocks (Models a-e)*.

Regarding illness perception, a greater sum score of clinician-rated PTSD symptoms at 3-month follow-up was significantly associated with item 1 “consequences” in the adjusted model (d). After taking into account all covariates, “consequences” explained 6 % of the total variance. The final model e showed no significant intervention-by-consequences interaction. Moreover, “consequences” was the only Brief IPQ dimension that showed a significant and independent association with PTSD symptoms.

Similar results were obtained with self-rated PTSD symptoms per the PDS sum score as the dependent variable (model output not shown). Specifically, demographic variables, fear of dying, and illness severity showed no significant associations in the final model (e). The intervention was not significantly linked to the PDS sum score in any model. Moreover, the intervention-by-consequences interaction did not emerge as a significant correlate of self-rated PTSD symptoms (model e). However, the level of depressive symptoms showed an independent and significant association with self-rated PTSD symptoms in all models, explaining 9% of the variance of the PDS sum score. Also, Item 1 “consequences” remained an independent correlate of the PDS sum score in the adjusted model (d), explaining 6% of the variance.

The adjusted R^2^ value for the adjusted models was 0.21 for the CAPS sum score and 0.28 for the PDS sum score, indicating that about 25% of the variance in clinician- and self-rated PTSD symptom sum scores was accounted for by the entire set of covariates.

## Discussion

Significant associations were found between illness perceptions assessed within 48 h of an acute MI and PTSD symptoms assessed 3 months later. Seven out of eight general illness perception factors were significantly associated with the development of PTSD symptomatology. These findings are consistent with the report of Sheldrick et al. (2006) who found illness perception factors of identity, timeline (acute/chronic), consequences, and emotional representation to be significant predictors of self-reported PTSD symptoms 3 months after acute MI. Oflaz (2014) found that illness perception factors of consequences, identity, concern and emotions were positively correlated with a PTSD diagnosis. Thus, illness perception of MI seems to impact on the development of PTSD after acute MI.

The main finding of this study was that beliefs about “consequences” were the most reliable predictor amongst the Brief IPQ variables. The result is consistent with the finding of Petrie et al. (1996) showing that the belief about illness consequences had a major impact on functioning outside work in post-MI patients. Moreover, these authors showed that an in-hospital intervention after MI to change patients' illness perceptions resulted in better functional outcome. However, Sheldrick et al. (2006) reported that among the illness perception factors showing correlations with PTSD, identity, timeline (acute/chronic), consequences and emotional representation, the latter was the most significant predictor of PTSD after MI and subarachnoid hemorrhage. Thus, beliefs about physical impairments as a consequence of illness and emotional responses to illness are both related to functioning in the wake of recovering from acute MI.

Additionally, a number of variables assessed at admission (i.e., depressive symptoms, fear of dying and CK-MB) showed bivariate correlations with PTSD symptoms. In addition to consequences, depressive symptoms emerged as a significant predictor for the sum score of self-rated PTSD symptoms at 3-month follow-up; this is in agreement with previous findings (Frasure-Smith et al., 1993). Interestingly, depressive symptoms and consequences explained a similar amount of variance in self-rated PTSD symptoms, while consequences, but not depressive symptoms, were significantly related to clinician-rated PTSD symptoms. One explanation for this observation could be that the CAPS interview specifically asks for MI-triggered traumatic symptoms making it possible to distinguish symptoms due to the traumatic sequel of MI from those better explained by other diseases such as heart failure and depression. Overall, the results underscore the importance of illness perception as a significant correlate of PTSD symptoms at 3-month follow-up across self-rated and clinician-rated PTSD symptomatology in post-MI patients.

We further found that the illness perception of older patients differed from that of younger ones. Particularly, younger patients showed higher illness concerns and assumed a longer illness time-line than older ones. A possible explanation for these difference might be that older patients have greater awareness of the possibility of becoming sick in the life course and, consequently, are more likely to accept the illness. Younger patients instead might feel substantially impaired in resuming responsibilities in daily life such as related to work, taking care of children and plans for the future. However, these assumptions are hypothetical and would need to be tested.

The prevalence of clinically relevant MI-induced PTSD symptoms is about 16% when using self-rated questionnaires, whereas 4% of patients can be diagnosed with full PTSD when applying a clinical interview (Edmondson et al., 2012). We found a slightly higher prevalence of clinically relevant PTSD symptoms based on self-ratings (18.8%), but a clearly lower prevalence of clinician-rated full PTSD (1%). The latter seems remarkable, as we only enrolled patients with a substantial amount of distress indexed by pain, fear of dying, and helplessness during MI. A higher prevalence might have been prevented in the present study as a counseling intervention was implemented with the aim to reduce the incidence of PTSD symptoms after acute MI as previously discussed (von Känel et al., 2018). However, as shown in the present study, and elaborated elsewhere (von Känel et al., 2018), the trauma-focused intervention was not more effective in reducing clinician-rated PTSD symptoms than the active control intervention targeting the general role of psychosocial stress in heart disease. Nonetheless, psychotherapeutic interventions implementing illness perceptions have been shown to be predictive for better recovery in terms of anxiety and returning to work after acute MI (Broadbent et al., 2009a). Therefore, a counseling session specifically targeting illness perceptions could perhaps help to substantially reduce the development of PTSD symptoms post-MI, although this needs to be investigated in future studies.

We assessed objective markers of MI severity to explore potential associations with PTSD symptoms at three-month follow-up. Although peak CK-MB values (but not the prognostic GRACE score) were significantly associated with PTSD symptoms in the bivariate analysis, CK-MB and the GRACE score did not emerge as independent predictors in the final covariate-adjusted model. These results suggest that illness beliefs, rather than the extent of heart muscle damage as an objective measure of illness severity, affect psychological adaptation to acute MI. This reasoning is consistent with previous investigations showing objective markers of myocardial damage were not predictors of PTSD symptoms (Guler et al., 2009; Edmondson and von Kanel, 2017).

## Limitations

Our study has several limitations. The sample size was small, which must be taken into account when interpreting the non-significant associations with several illness perception subscales. We only assessed patients who were referred to a tertiary university hospital and perceived high level of distress during MI, so the generalizability of our findings is limited. We assessed the Brief IPQ at admission only; that is very shortly after MI. Therefore, modification of illness perceptions over the course of the disease was not evaluated. Furthermore, a better comprehension of the role of illness representation would have been captured using the extended IPQ questionnaire. Exclusion of patients with severe depression likely resulted in a small variance of depressive symptoms at admission, a possible explanation for why depressive symptoms did not predict PTSD symptoms any stronger than illness perceptions. Also, it must be noted that the data analyzed for the present study were collected as part of a randomized behavioral intervention trial aimed at reducing the incidence of clinician rated PTSD symptoms (Meister et al., 2013). In addition, it is possible that another variable, such as social support, could account for some of the association between illness perception and PTSD symptoms.

## Conclusions

This study highlights the importance of illness perception and its assessment as a valuable tool in identifying patients likely to develop clinically relevant PTSD symptoms post-MI. Our data suggest that illness perception may play a more important role in the manifestation of PTSD symptoms after MI than do objective markers of MI severity or demographic variables. Cognitive depressive symptoms were also strongly associated with self-rated PTSD symptoms and may enhance negative illness perception, although negative illness perception might also enhance depressive cognitions. Therefore, prevention strategies targeting illness perceptions, but also cognitive depressive symptoms after acute MI, might decrease the risk of developing clinically relevant PTSD symptoms. To reduce PTSD symptoms in patients after acute MI, we would recommend psychological interventions targeting negative illness perceptions while patients are still in the hospital. Medical staff awareness should be trained. Future trials are needed to test such interventions and their effects on PTSD symptoms as an important outcome after acute MI. Taken together, the present study showed that illness perceptions were independently associated with the development of PTSD symptoms attributable to acute MI.

## Ethics statement

All procedures performed in studies involving human participants were in accordance with the ethical standards of the institutional and national research committee and with the 1964 Helsinki declaration and its later amendments or comparable ethical standards. The study protocol was approved by the ethics committee of the State of Bern, Switzerland.

## Informed consent

Informed consent was obtained from all individual participants included in the study.

## Author contributions

RvK, J-PS, US, JB, and HZ contributed to the development of the study design. US, HZ, JB, RvK, RM-L, and MP contributed to the development of the verum and control intervention. MP and CG wrote the first draft of the manuscript. All authors critically revised and approved the final manuscript.

### Conflict of interest statement

The authors declare that the research was conducted in the absence of any commercial or financial relationships that could be construed as a potential conflict of interest.

## Abbreviations

BDI-II: The Beck Depression Inventory second edition
CAPS: Clinician-Administered PTSD Scale
CK-MB: Creatine kinase isoenzyme MB
CCU: Coronary Care Unit
DSM-IV: Diagnostic and Statistical Manual of Mental Disorders fourth edition
DSM-5: Diagnostic and Statistical Manual of Mental Disorders fifth edition
GRACE score: Global Registry of Acute Coronary Events (GRACE) score
Brief IPQ: Brief Illness Perception Questionnaire
MI: Myocardial infarction
MI-SPRINT: MI-SPRINT: Myocardial Infarction—Stress PRevention INTervention
NSTEMI: non-ST-segment elevation myocardial infarction
PDS: Self-report Posttraumatic Diagnostic Scale
PTSD: Posttraumatic Stress Disorder
STEMI: ST-segment elevation myocardial infarction.
